# 4-{[1-(4-Ethoxy­phen­yl)-5-methyl-1*H*-1,2,3-triazol-4-yl]diphenyl­meth­yl}morpholine

**DOI:** 10.1107/S1600536808012725

**Published:** 2008-05-14

**Authors:** Jian-Guo Wu, Hong-Ru Dong, Heng-Shan Dong, Seik Weng Ng

**Affiliations:** a State Key Laboratory of Applied Organic Chemistry, Institute of Organic Chemistry, College of Chemistry and Chemical Engineering, Lanzhou University, Lanzhou, Gansu 730000, People’s Republic of China; b Department of Chemistry, University of Malaya, 50603 Kuala Lumpur, Malaysia

## Abstract

The title compound, C_28_H_30_N_4_O_2_, synthesized from 4-[1-(4-ethoxy­phen­yl)-5-methyl-1*H*-1,2,3-triazol-4-yl]diphenyl­meth­an­ol and morpholine, consists of a subsituted triazolyl group and a morpholinyl group that crowd the aliphatic C atom of a diphenyl­methyl unit [C_triaz_—C—N_morph_ = 110.1 (1)° and C_phen­yl_—C—C_phen­yl_ = 103.9 (1)°]. The morpholine ring adopts a chair conformation.

## Related literature

For background literature on the synthesis of the precursor (1-aryl-5-methyl-1*H*-1,2,3-triazol-4-yl)diaryl­methanols, see: Dong *et al.* (2008 ▶).
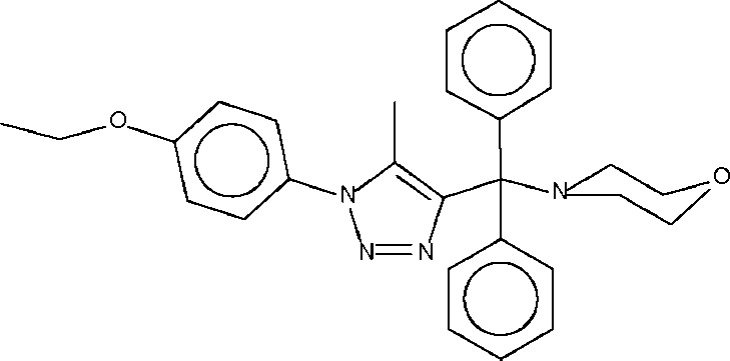


## Experimental

### 

#### Crystal data

C_28_H_30_N_4_O_2_
*M* *_r_* = 454.56
Triclinic, 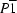

*a* = 9.406 (1) Å
*b* = 10.125 (1) Å
*c* = 13.670 (2) Å
α = 81.408 (1)°
β = 73.621 (1)°
γ = 81.547 (1)°
*V* = 1227.4 (2) Å^3^
*Z* = 2
Mo *K*α radiation
μ = 0.08 mm^−1^
*T* = 294 (2) K
0.28 × 0.25 × 0.20 mm

#### Data collection

Bruker APEXII diffractometer
Absorption correction: none
6395 measured reflections
4437 independent reflections
2830 reflections with *I* > 2σ(*I*)
*R*_int_ = 0.019

#### Refinement

*R*[*F* ^2^ > 2σ(*F* ^2^)] = 0.046
*wR*(*F* ^2^) = 0.126
*S* = 0.99
4437 reflections
309 parameters
H-atom parameters constrained
Δρ_max_ = 0.14 e Å^−3^
Δρ_min_ = −0.23 e Å^−3^

### 

Data collection: *APEX2* (Bruker, 2004 ▶); cell refinement: *SAINT* (Bruker, 2004 ▶); data reduction: *SAINT*; program(s) used to solve structure: *SHELXS97* (Sheldrick, 2008 ▶); program(s) used to refine structure: *SHELXL97* (Sheldrick, 2008 ▶); molecular graphics: *X-SEED* (Barbour, 2001 ▶); software used to prepare material for publication: *publCIF* (Westrip, 2008 ▶).

## Supplementary Material

Crystal structure: contains datablocks I, global. DOI: 10.1107/S1600536808012725/hg2394sup1.cif

Structure factors: contains datablocks I. DOI: 10.1107/S1600536808012725/hg2394Isup2.hkl

Additional supplementary materials:  crystallographic information; 3D view; checkCIF report

## Figures and Tables

**Fig. 1.**
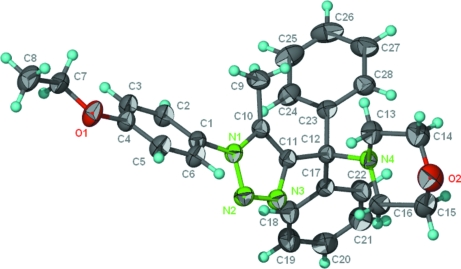
50% Probability thermal ellipsoid plot (Barbour, 2001) of C28H30N4O2. Hydrogen atoms are drawn as spheres of arbitrary radii.

## supplementary crystallographic information

### Comment

We have recently reported the synthesis of some (1-aryl-5-methyl-1*H*-1,2,3-triazol-4-yl)diarylmethanols and characterized one of them, 1-(4-tolyl)-5-methyl-1*H*-1,2,3-triazol-4-yl]bis(3-chlorophenyl)methanol, by X-ray crystallography (Dong *et al.*, 2008). In the present study, the methanolic –OH group of [1-(4-ethoxyphenyl)-5-methyl-1*H*-1,2,3-triazol-4-yl]diphenylmethanol is replaced by a morpholinyl ring in the expectation that the resulting compound (Scheme I) will possess enhanced biological activity. The compound, C_28_H_30_N_4_O_2 _(Fig. 1), consists of a subsituted triazolyl part and a morpholinyl part that crowd the aliphatic carbon atom of the diphenylmethyl entity, the crowding depressing the C_phenyl_–*C*–C_phenyl_ angle [103.9 (1)°] from the idealized angle. The morpholinyl ring adopts a chair conformation.

### Experimental

[1-(4-Ethoxyphenyl)-5-methyl-1*H*-1,2,3-triazol-4-yl]diphenylmethanol, which was synthesized by a modification of a published procedure (Dong *et al.*, 2008) (1.2 g, 3.2 mmol), was dissolved in benzene (30 ml); dry hydrogen chloride gas was passed into the refluxing solution until the theoretical quantity of water was formed. Morpholine (0.4 ml) and triethylamine (0.7 ml) were added and the mixture kept at 318 K for two hours. Removal of the solvent gave a solid; this was washed with water, dried and recrystallized from ethyl acetate to give the pure compound, m.p. 456–458 K in 90% yield. The formulation was established by ^1^H-NMR and mass spectrosopic analyses. ^1^H-NMR(300 MHz, CDCl_3_): 1.422–1.469 (t, 3H, *J *= 6.9 Hz, ArOCH_2_–CH_3_), 2.049 (s, 3H, triazolyl–CH_3_), 2.456 (br, 4H, –N(CH_2_)_2_–), 3.829–3.859 (t, 4H, *J *= 4.5 Hz, –CH_2_OCH_2_–), 4.044–4.114 (q, 2H, *J *= 6.9 Hz, ArO–CH_2_–), 6.975–7.006 (d, 2H, *J *= 9.3 Hz, C_2_H_5_OAr–3,5*H*), 7.143–7.192 (t, 2H, *J *= 7.5 Hz, Ar–4H), 7.258–7.352 (m, 6H, C_2_H_5_OAr–2,6*H*, Ar–3,5*H*), 7.557–7.583 (d, 4H, *J *= 7.8 Hz, Ar–2,6*H*) p.p.m.. MS (%): 454 (*M*^+.^, 0.88%), 369 (68), 340 (59), 312 (17), 310 (13), 264 (7.3), 252 (7.1), 224 (4.8), 219 (3.1), 205 (9.9), 191 (6.7), 178 (18), 165 (15), 162 (100), 151 (16), 149 (40), 134 (19), 121 (12), 93 (11), 91 (16), 77 (17).

### Refinement

Carbon-bound H-atoms were placed in calculated positions (C—H 0.93 to 0.98 Å) and were included in the refinement in the riding model approximation, with *U*(H) set to 1.2 to 1.5*U*_eq_(C). The methyl groups were rotated to fit the electron density.

### Crystal data

**Table:**

C_28_H_30_N_4_O_2_	*Z* = 2
*M**_r_* = 454.56	*F*_000_ = 484
Triclinic, *P*1	*D*_x_ = 1.230 Mg m^−^^3^
Hall symbol: -P 1	Melting point: 457 K
*a* = 9.406 (1) Å	Mo *K*α radiation λ = 0.71073 Å
*b* = 10.125 (1) Å	Cell parameters from 1386 reflections
*c* = 13.670 (2) Å	θ = 2.3–22.5º
α = 81.408 (1)º	µ = 0.08 mm^−^^1^
β = 73.621 (1)º	*T* = 294 (2) K
γ = 81.547 (1)º	Rhombohedron, colorless
*V* = 1227.4 (2) Å^3^	0.28 × 0.25 × 0.20 mm

### Data collection

**Table:**

Bruker APEXII diffractometer	2830 reflections with *I* > 2σ(*I*)
Radiation source: fine-focus sealed tube	*R*_int_ = 0.019
Monochromator: graphite	θ_max_ = 25.0º
*T* = 295(2) K	θ_min_ = 2.0º
φ and ω scans	*h* = −7→11
Absorption correction: None	*k* = −9→12
6395 measured reflections	*l* = −16→16
4437 independent reflections	

### Refinement

**Table:**

Refinement on *F*^2^	Secondary atom site location: difference Fourier map
Least-squares matrix: full	Hydrogen site location: inferred from neighbouring sites
*R*[*F*^2^ > 2σ(*F*^2^)] = 0.046	H-atom parameters constrained
*wR*(*F*^2^) = 0.126	*w* = 1/[σ^2^(*F*_o_^2^) + (0.0614*P*)^2^] where *P* = (*F*_o_^2^ + 2*F*_c_^2^)/3
*S* = 0.99	(Δ/σ)_max_ = 0.001
4437 reflections	Δρ_max_ = 0.14 e Å^−^^3^
309 parameters	Δρ_min_ = −0.23 e Å^−^^3^
Primary atom site location: structure-invariant direct methods	Extinction correction: none

### Fractional atomic coordinates and isotropic or equivalent isotropic displacement parameters (Å^2^)

**Table:**

	*x*	*y*	*z*	*U*_iso_*/*U*_eq_	
O1	−0.01051 (14)	0.78624 (14)	0.99479 (11)	0.0597 (4)	
O2	1.10986 (17)	0.31482 (19)	0.68284 (13)	0.0761 (5)	
N1	0.43338 (16)	0.39052 (15)	0.83355 (11)	0.0395 (4)	
N2	0.49124 (17)	0.30714 (16)	0.90338 (12)	0.0462 (4)	
N3	0.59072 (17)	0.21953 (16)	0.85227 (11)	0.0447 (4)	
N4	0.86454 (15)	0.19704 (15)	0.66019 (11)	0.0392 (4)	
C1	0.31717 (19)	0.49423 (18)	0.86992 (14)	0.0391 (5)	
C2	0.1836 (2)	0.5083 (2)	0.84476 (14)	0.0468 (5)	
H2	0.1686	0.4521	0.8015	0.056*	
C3	0.0710 (2)	0.6066 (2)	0.88424 (15)	0.0479 (5)	
H3	−0.0188	0.6173	0.8665	0.057*	
C4	0.0926 (2)	0.68869 (19)	0.94988 (14)	0.0428 (5)	
C5	0.2269 (2)	0.6723 (2)	0.97548 (15)	0.0495 (5)	
H5	0.2415	0.7266	1.0202	0.059*	
C6	0.3386 (2)	0.57633 (19)	0.93530 (15)	0.0458 (5)	
H6	0.4290	0.5666	0.9522	0.055*	
C7	−0.1432 (2)	0.8214 (2)	0.96182 (15)	0.0538 (6)	
H7A	−0.2016	0.7458	0.9783	0.065*	
H7B	−0.1189	0.8464	0.8882	0.065*	
C8	−0.2296 (3)	0.9376 (2)	1.01669 (19)	0.0735 (7)	
H8A	−0.3161	0.9686	0.9920	0.110*	
H8B	−0.1679	1.0092	1.0042	0.110*	
H8C	−0.2602	0.9095	1.0891	0.110*	
C9	0.4629 (2)	0.4427 (2)	0.64574 (15)	0.0570 (6)	
H9A	0.4425	0.5350	0.6592	0.086*	
H9B	0.3771	0.4150	0.6329	0.086*	
H9C	0.5466	0.4334	0.5867	0.086*	
C10	0.49787 (19)	0.35671 (18)	0.73669 (14)	0.0389 (5)	
C11	0.59709 (19)	0.24503 (18)	0.75005 (14)	0.0372 (4)	
C12	0.71084 (18)	0.15980 (18)	0.67455 (13)	0.0365 (4)	
C13	0.8762 (2)	0.3413 (2)	0.63607 (17)	0.0545 (6)	
H13A	0.8234	0.3869	0.6957	0.065*	
H13B	0.8308	0.3771	0.5807	0.065*	
C14	1.0384 (2)	0.3658 (2)	0.60466 (18)	0.0663 (7)	
H14A	1.0893	0.3234	0.5432	0.080*	
H14B	1.0454	0.4617	0.5885	0.080*	
C15	1.0936 (2)	0.1761 (3)	0.71158 (19)	0.0702 (7)	
H15A	1.1395	0.1434	0.7672	0.084*	
H15B	1.1456	0.1267	0.6538	0.084*	
C16	0.9328 (2)	0.1497 (2)	0.74525 (16)	0.0557 (6)	
H16A	0.9261	0.0542	0.7647	0.067*	
H16B	0.8805	0.1964	0.8044	0.067*	
C17	0.70954 (19)	0.00784 (18)	0.71035 (13)	0.0381 (4)	
C18	0.5995 (2)	−0.0457 (2)	0.78943 (16)	0.0498 (5)	
H18	0.5237	0.0118	0.8264	0.060*	
C19	0.5992 (2)	−0.1829 (2)	0.81513 (17)	0.0607 (6)	
H19	0.5233	−0.2163	0.8689	0.073*	
C20	0.7093 (3)	−0.2706 (2)	0.76228 (17)	0.0586 (6)	
H20	0.7092	−0.3629	0.7800	0.070*	
C21	0.8192 (3)	−0.2191 (2)	0.68285 (17)	0.0607 (6)	
H21	0.8944	−0.2771	0.6460	0.073*	
C22	0.8196 (2)	−0.0820 (2)	0.65696 (16)	0.0531 (5)	
H22	0.8952	−0.0491	0.6027	0.064*	
C23	0.6762 (2)	0.17581 (18)	0.56946 (14)	0.0398 (5)	
C24	0.5331 (2)	0.1628 (2)	0.56461 (17)	0.0549 (6)	
H24	0.4577	0.1505	0.6250	0.066*	
C25	0.5019 (3)	0.1679 (2)	0.4711 (2)	0.0690 (7)	
H25	0.4054	0.1605	0.4691	0.083*	
C26	0.6119 (3)	0.1836 (2)	0.3817 (2)	0.0746 (8)	
H26	0.5905	0.1873	0.3189	0.089*	
C27	0.7536 (3)	0.1939 (2)	0.38524 (17)	0.0708 (7)	
H27	0.8290	0.2035	0.3245	0.085*	
C28	0.7858 (2)	0.1903 (2)	0.47848 (15)	0.0534 (6)	
H28	0.8826	0.1977	0.4797	0.064*	

### Atomic displacement parameters (Å^2^)

**Table:**

	*U*^11^	*U*^22^	*U*^33^	*U*^12^	*U*^13^	*U*^23^
O1	0.0467 (9)	0.0636 (10)	0.0720 (10)	0.0161 (7)	−0.0198 (7)	−0.0315 (8)
O2	0.0700 (11)	0.0936 (14)	0.0784 (12)	−0.0350 (9)	−0.0273 (9)	−0.0124 (10)
N1	0.0391 (9)	0.0370 (9)	0.0405 (9)	0.0024 (7)	−0.0088 (7)	−0.0084 (7)
N2	0.0482 (10)	0.0465 (10)	0.0431 (9)	0.0064 (8)	−0.0137 (8)	−0.0107 (8)
N3	0.0453 (10)	0.0457 (10)	0.0420 (9)	0.0050 (7)	−0.0114 (8)	−0.0120 (8)
N4	0.0350 (9)	0.0435 (10)	0.0399 (9)	−0.0056 (7)	−0.0097 (7)	−0.0063 (7)
C1	0.0363 (11)	0.0372 (11)	0.0418 (11)	0.0000 (8)	−0.0074 (8)	−0.0080 (9)
C2	0.0460 (12)	0.0499 (13)	0.0486 (12)	−0.0020 (9)	−0.0143 (10)	−0.0184 (10)
C3	0.0385 (11)	0.0556 (14)	0.0518 (12)	−0.0004 (9)	−0.0143 (9)	−0.0139 (10)
C4	0.0386 (11)	0.0416 (12)	0.0458 (11)	0.0027 (8)	−0.0075 (9)	−0.0115 (9)
C5	0.0497 (12)	0.0472 (13)	0.0580 (13)	0.0045 (9)	−0.0210 (10)	−0.0230 (10)
C6	0.0398 (11)	0.0442 (12)	0.0574 (13)	−0.0011 (9)	−0.0173 (9)	−0.0140 (10)
C7	0.0433 (12)	0.0603 (15)	0.0537 (13)	0.0073 (10)	−0.0133 (10)	−0.0062 (11)
C8	0.0607 (15)	0.0736 (18)	0.0824 (17)	0.0250 (12)	−0.0215 (13)	−0.0245 (14)
C9	0.0676 (14)	0.0489 (14)	0.0469 (12)	0.0082 (10)	−0.0117 (10)	−0.0025 (10)
C10	0.0394 (11)	0.0376 (11)	0.0374 (11)	−0.0010 (8)	−0.0064 (8)	−0.0072 (9)
C11	0.0357 (10)	0.0387 (11)	0.0381 (11)	−0.0021 (8)	−0.0101 (8)	−0.0088 (8)
C12	0.0343 (10)	0.0371 (11)	0.0368 (10)	−0.0013 (8)	−0.0071 (8)	−0.0075 (8)
C13	0.0529 (13)	0.0483 (14)	0.0624 (14)	−0.0114 (10)	−0.0118 (11)	−0.0078 (11)
C14	0.0630 (15)	0.0685 (17)	0.0713 (16)	−0.0269 (12)	−0.0161 (13)	−0.0038 (13)
C15	0.0514 (14)	0.093 (2)	0.0739 (16)	−0.0184 (13)	−0.0269 (12)	−0.0018 (15)
C16	0.0476 (13)	0.0729 (16)	0.0506 (13)	−0.0136 (10)	−0.0192 (10)	−0.0004 (11)
C17	0.0371 (10)	0.0389 (11)	0.0397 (11)	−0.0016 (8)	−0.0124 (9)	−0.0072 (9)
C18	0.0435 (12)	0.0436 (13)	0.0573 (13)	−0.0032 (9)	−0.0037 (10)	−0.0107 (10)
C19	0.0583 (14)	0.0498 (15)	0.0693 (16)	−0.0149 (11)	−0.0071 (12)	−0.0021 (12)
C20	0.0746 (16)	0.0380 (13)	0.0671 (15)	−0.0085 (11)	−0.0260 (13)	−0.0023 (11)
C21	0.0698 (15)	0.0442 (14)	0.0623 (14)	0.0079 (11)	−0.0113 (12)	−0.0139 (11)
C22	0.0539 (13)	0.0446 (13)	0.0516 (13)	0.0006 (9)	−0.0016 (10)	−0.0065 (10)
C23	0.0460 (11)	0.0324 (11)	0.0425 (11)	0.0013 (8)	−0.0145 (9)	−0.0090 (8)
C24	0.0554 (14)	0.0533 (14)	0.0637 (14)	−0.0053 (10)	−0.0261 (11)	−0.0110 (11)
C25	0.0843 (18)	0.0559 (16)	0.0863 (19)	−0.0046 (12)	−0.0538 (16)	−0.0121 (13)
C26	0.120 (2)	0.0568 (16)	0.0623 (17)	0.0066 (15)	−0.0526 (17)	−0.0146 (13)
C27	0.096 (2)	0.0720 (18)	0.0433 (13)	0.0057 (14)	−0.0225 (13)	−0.0090 (12)
C28	0.0586 (14)	0.0582 (14)	0.0418 (12)	0.0035 (10)	−0.0134 (10)	−0.0101 (10)

### Geometric parameters (Å, °)

**Table:**

O1—C4	1.363 (2)	C12—C23	1.541 (2)
O1—C7	1.424 (2)	C12—C17	1.544 (2)
O2—C14	1.414 (3)	C13—C14	1.512 (3)
O2—C15	1.419 (3)	C13—H13A	0.9700
N1—N2	1.3554 (19)	C13—H13B	0.9700
N1—C10	1.364 (2)	C14—H14A	0.9700
N1—C1	1.434 (2)	C14—H14B	0.9700
N2—N3	1.3126 (19)	C15—C16	1.504 (3)
N3—C11	1.368 (2)	C15—H15A	0.9700
N4—C13	1.462 (2)	C15—H15B	0.9700
N4—C16	1.469 (2)	C16—H16A	0.9700
N4—C12	1.500 (2)	C16—H16B	0.9700
C1—C2	1.376 (2)	C17—C18	1.376 (3)
C1—C6	1.379 (3)	C17—C22	1.388 (2)
C2—C3	1.390 (2)	C18—C19	1.381 (3)
C2—H2	0.9300	C18—H18	0.9300
C3—C4	1.383 (3)	C19—C20	1.372 (3)
C3—H3	0.9300	C19—H19	0.9300
C4—C5	1.384 (2)	C20—C21	1.370 (3)
C5—C6	1.372 (2)	C20—H20	0.9300
C5—H5	0.9300	C21—C22	1.381 (3)
C6—H6	0.9300	C21—H21	0.9300
C7—C8	1.499 (3)	C22—H22	0.9300
C7—H7A	0.9700	C23—C28	1.378 (3)
C7—H7B	0.9700	C23—C24	1.392 (3)
C8—H8A	0.9600	C24—C25	1.382 (3)
C8—H8B	0.9600	C24—H24	0.9300
C8—H8C	0.9600	C25—C26	1.366 (3)
C9—C10	1.495 (2)	C25—H25	0.9300
C9—H9A	0.9600	C26—C27	1.367 (3)
C9—H9B	0.9600	C26—H26	0.9300
C9—H9C	0.9600	C27—C28	1.385 (3)
C10—C11	1.381 (2)	C27—H27	0.9300
C11—C12	1.525 (2)	C28—H28	0.9300
			
C4—O1—C7	118.54 (15)	N4—C13—H13A	109.7
C14—O2—C15	110.11 (17)	C14—C13—H13A	109.7
N2—N1—C10	111.22 (14)	N4—C13—H13B	109.7
N2—N1—C1	118.18 (14)	C14—C13—H13B	109.7
C10—N1—C1	130.58 (15)	H13A—C13—H13B	108.2
N3—N2—N1	106.67 (14)	O2—C14—C13	111.94 (19)
N2—N3—C11	109.81 (14)	O2—C14—H14A	109.2
C13—N4—C16	107.06 (16)	C13—C14—H14A	109.2
C13—N4—C12	113.90 (13)	O2—C14—H14B	109.2
C16—N4—C12	116.16 (14)	C13—C14—H14B	109.2
C2—C1—C6	120.09 (17)	H14A—C14—H14B	107.9
C2—C1—N1	121.16 (16)	O2—C15—C16	112.19 (19)
C6—C1—N1	118.67 (16)	O2—C15—H15A	109.2
C1—C2—C3	119.83 (18)	C16—C15—H15A	109.2
C1—C2—H2	120.1	O2—C15—H15B	109.2
C3—C2—H2	120.1	C16—C15—H15B	109.2
C4—C3—C2	119.99 (18)	H15A—C15—H15B	107.9
C4—C3—H3	120.0	N4—C16—C15	108.98 (17)
C2—C3—H3	120.0	N4—C16—H16A	109.9
O1—C4—C3	125.11 (17)	C15—C16—H16A	109.9
O1—C4—C5	115.37 (17)	N4—C16—H16B	109.9
C3—C4—C5	119.51 (17)	C15—C16—H16B	109.9
C6—C5—C4	120.41 (18)	H16A—C16—H16B	108.3
C6—C5—H5	119.8	C18—C17—C22	117.13 (18)
C4—C5—H5	119.8	C18—C17—C12	123.62 (16)
C5—C6—C1	120.16 (18)	C22—C17—C12	119.13 (17)
C5—C6—H6	119.9	C17—C18—C19	121.45 (18)
C1—C6—H6	119.9	C17—C18—H18	119.3
O1—C7—C8	107.35 (17)	C19—C18—H18	119.3
O1—C7—H7A	110.2	C20—C19—C18	120.8 (2)
C8—C7—H7A	110.2	C20—C19—H19	119.6
O1—C7—H7B	110.2	C18—C19—H19	119.6
C8—C7—H7B	110.2	C21—C20—C19	118.5 (2)
H7A—C7—H7B	108.5	C21—C20—H20	120.7
C7—C8—H8A	109.5	C19—C20—H20	120.7
C7—C8—H8B	109.5	C20—C21—C22	120.7 (2)
H8A—C8—H8B	109.5	C20—C21—H21	119.6
C7—C8—H8C	109.5	C22—C21—H21	119.6
H8A—C8—H8C	109.5	C21—C22—C17	121.3 (2)
H8B—C8—H8C	109.5	C21—C22—H22	119.3
C10—C9—H9A	109.5	C17—C22—H22	119.3
C10—C9—H9B	109.5	C28—C23—C24	117.90 (18)
H9A—C9—H9B	109.5	C28—C23—C12	122.05 (17)
C10—C9—H9C	109.5	C24—C23—C12	119.77 (17)
H9A—C9—H9C	109.5	C25—C24—C23	120.8 (2)
H9B—C9—H9C	109.5	C25—C24—H24	119.6
N1—C10—C11	104.16 (15)	C23—C24—H24	119.6
N1—C10—C9	120.80 (16)	C26—C25—C24	120.4 (2)
C11—C10—C9	134.81 (17)	C26—C25—H25	119.8
N3—C11—C10	108.11 (15)	C24—C25—H25	119.8
N3—C11—C12	119.18 (15)	C25—C26—C27	119.5 (2)
C10—C11—C12	132.53 (16)	C25—C26—H26	120.2
N4—C12—C11	110.13 (14)	C27—C26—H26	120.2
N4—C12—C23	109.34 (14)	C26—C27—C28	120.5 (2)
C11—C12—C23	112.04 (13)	C26—C27—H27	119.7
N4—C12—C17	108.86 (13)	C28—C27—H27	119.7
C11—C12—C17	112.32 (14)	C23—C28—C27	120.8 (2)
C23—C12—C17	103.93 (14)	C23—C28—H28	119.6
N4—C13—C14	109.61 (16)	C27—C28—H28	119.6
			
C10—N1—N2—N3	−0.7 (2)	C10—C11—C12—C23	−20.6 (3)
C1—N1—N2—N3	177.98 (16)	N3—C11—C12—C17	48.4 (2)
N1—N2—N3—C11	−0.4 (2)	C10—C11—C12—C17	−137.1 (2)
N2—N1—C1—C2	−126.47 (19)	C16—N4—C13—C14	60.2 (2)
C10—N1—C1—C2	51.9 (3)	C12—N4—C13—C14	−169.95 (15)
N2—N1—C1—C6	50.3 (2)	C15—O2—C14—C13	55.7 (2)
C10—N1—C1—C6	−131.3 (2)	N4—C13—C14—O2	−59.1 (2)
C6—C1—C2—C3	0.9 (3)	C14—O2—C15—C16	−56.5 (2)
N1—C1—C2—C3	177.65 (17)	C13—N4—C16—C15	−60.5 (2)
C1—C2—C3—C4	−1.0 (3)	C12—N4—C16—C15	170.98 (17)
C7—O1—C4—C3	−9.5 (3)	O2—C15—C16—N4	59.9 (2)
C7—O1—C4—C5	171.69 (18)	N4—C12—C17—C18	134.58 (18)
C2—C3—C4—O1	−178.55 (18)	C11—C12—C17—C18	12.3 (2)
C2—C3—C4—C5	0.2 (3)	C23—C12—C17—C18	−108.97 (19)
O1—C4—C5—C6	179.58 (18)	N4—C12—C17—C22	−49.4 (2)
C3—C4—C5—C6	0.7 (3)	C11—C12—C17—C22	−171.63 (16)
C4—C5—C6—C1	−0.8 (3)	C23—C12—C17—C22	67.1 (2)
C2—C1—C6—C5	0.0 (3)	C22—C17—C18—C19	0.5 (3)
N1—C1—C6—C5	−176.83 (17)	C12—C17—C18—C19	176.55 (18)
C4—O1—C7—C8	−175.31 (18)	C17—C18—C19—C20	0.0 (3)
N2—N1—C10—C11	1.4 (2)	C18—C19—C20—C21	−0.4 (3)
C1—N1—C10—C11	−177.04 (18)	C19—C20—C21—C22	0.3 (3)
N2—N1—C10—C9	−173.86 (17)	C20—C21—C22—C17	0.2 (3)
C1—N1—C10—C9	7.7 (3)	C18—C17—C22—C21	−0.6 (3)
N2—N3—C11—C10	1.3 (2)	C12—C17—C22—C21	−176.84 (18)
N2—N3—C11—C12	177.05 (16)	N4—C12—C23—C28	14.1 (2)
N1—C10—C11—N3	−1.6 (2)	C11—C12—C23—C28	136.44 (19)
C9—C10—C11—N3	172.7 (2)	C17—C12—C23—C28	−102.1 (2)
N1—C10—C11—C12	−176.61 (18)	N4—C12—C23—C24	−172.18 (15)
C9—C10—C11—C12	−2.3 (4)	C11—C12—C23—C24	−49.8 (2)
C13—N4—C12—C11	−50.70 (19)	C17—C12—C23—C24	71.71 (19)
C16—N4—C12—C11	74.41 (19)	C28—C23—C24—C25	−1.7 (3)
C13—N4—C12—C23	72.82 (18)	C12—C23—C24—C25	−175.68 (18)
C16—N4—C12—C23	−162.07 (15)	C23—C24—C25—C26	1.1 (3)
C13—N4—C12—C17	−174.25 (15)	C24—C25—C26—C27	0.2 (4)
C16—N4—C12—C17	−49.1 (2)	C25—C26—C27—C28	−0.8 (3)
N3—C11—C12—N4	−73.2 (2)	C24—C23—C28—C27	1.0 (3)
C10—C11—C12—N4	101.4 (2)	C12—C23—C28—C27	174.90 (18)
N3—C11—C12—C23	164.90 (16)	C26—C27—C28—C23	0.2 (3)
